# Iridoid glycosides from *Morinda officinalis* How. exert anti-inflammatory and anti-arthritic effects through inactivating MAPK and NF-κB signaling pathways

**DOI:** 10.1186/s12906-020-02895-7

**Published:** 2020-06-05

**Authors:** Qi Zhang, Jian-hua Zhang, Yu-qiong He, Quan-long Zhang, Bo Zhu, Yi Shen, Meng-qin Liu, Lu-lin Zhu, Hai-liang Xin, Lu-ping Qin, Qiao-yan Zhang

**Affiliations:** 1 College of Pharmaceutical Sciences, Zhejiang University of Traditional Chinese Medicine, Gaoke Road, Fuyang District, Hangzhou, 310053 People’s Republic of China; 2 School of Pharmacy, Fujian University of Traditional Chinese Medicine, Fuzhou, 350122 China; 3 School of Pharmacy, Second Military Medical University, Shanghai, 200433 China

**Keywords:** *Morinda officinalis* how., Iridoid glycoside, Anti-inflammation, Anti-arthritis, RAW 264.7 macrophages, NF-κB, MAPK pathway

## Abstract

**Background:**

The root of *Morinda officinalis* How. (MO, the family of Rubiaceae) has long been used to treat inflammatory diseases in China and other eastern Asian countries, and iridoid glycosides extracted from MO (MOIG) are believed to contribute to this anti-inflammatory effect. However, the mechanism underlying the anti-inflammatory and anti-arthritic activities of MOIG has not been elucidated. The aim of the present study was to determine how MOIG exerted anti-inflammatory and anti-arthritic effects in vivo and in RAW 264.7 macrophages.

**Methods:**

MOIG were enriched by XDA-1 macroporous resin. The maximum feasible dose method was adopted to evaluate its acute toxicity. The analgesic effect of MOIG was evaluated by acetic acid writhing test and the anti-inflammatory effect was evaluated by cotton-pellet granuloma test in rats and air pouch granuloma test in mice. The anti-arthritic effect was evaluated by establishing an adjuvant arthritis model induced by Complete Freund’s Adjuvant (CFA). The viability of the cultured RAW 264.7 macrophages was assessed by 3-(4, 5-dimethylthiazol-2-yl)-2, 5-diphenyl tetrazolium bromide (MTT) assay. The anti-inflammatory activity was evaluated by measuring NO, IL-1β, IL-6 and TNF-α levels in LPS-stimulated RAW 264.7 cells. The protein level of inflammatory responsive genes was evaluated by Western blot analysis.

**Results:**

MOIG had no significant toxicity at maximum feasible dose of 22.5 g/kg. MO extracts and MOIG (50,100 and 200 mg/kg) all evoked a significantly inhibitory effects on the frequency of twisting induced by acetic acid in mice compared with the model control group. Administration of MO extracts and MOIG markedly decreased the dry and wet weight of cotton pellet granuloma in rats and air pouch granuloma in mice. MOIG significantly attenuated the paw swelling and decreased the arthritic score, weight loss, spleen index, and the serum level of inflammatory factors IL-1β, IL-6 and IL-17a in CFA-induced arthritic rats. MOIG inhibited the production of inflammatory cytokines in LPS-stimulated RAW264.7 cells, and the expressions of iNOS, COX-2 and proteins related to MAPK and NF-κB signaling pathways in LPS-stimulated RAW 264.7 macrophages.

**Conclusion:**

MOIG exerted anti-inflammatory and anti-arthritic activities through inactivating MAPK and NF-κB signaling pathways, and this finding may provide a sound experimental basis for the clinical treatment of rheumatoid arthritis with MOIG.

## Background

Rheumatoid arthritis (RA), a chronic and autoimmune disease, is featured with inflammatory cell infiltration and proliferation of synovial tissue, and eventually leading to bone deterioration [1]. Due to the rapid inducing inflammation effects on multisystem and irreversible joint damage, RA often resulted in premature mortality, disability and a low-quality of life in modern society [2]. Although the medical therapy for RA, such as disease-modifying anti-rheumatic drugs (DMARDs) supplemented with non-steroidal anti-inflammatory drugs (NSAIDs), steroid hormone and biologics, have achieved good therapeutic effects, some severe adverse reactions, including gastrointestinal tract reaction, cardiovascular complications and reproductive system toxicity, have aroused people’s concerns [3, 4], and also promoted people to pay more attention to anti-RA natural medicine with high efficacy and fewer adverse effects. Furthermore, it has been indicated that more than half of RA patients prefer to take natural medicines instead of synthetic chemicals [5]. Therefore, it is urgent to develop and explore safer and more effective anti-RA medicine derived from natural products.

*Morinda officinalis* How. (MO, the family of Rubiaceae), as potential medicinal herb, has been widely applied for prevention and treatment of various disease, inclusion of impotence, osteoporosis, rheumatoid arthritis, dermatitis, depression, and Alzheimer disease [6–9]. Phytochemical analysis indicates that a variety of chemical constituents exist in MO, including iridoid glycosides, anthraquinones, polysaccharides and oligosaccharides [7]. Of these chemical constituents, iridoid glycosides aroused our attention and interest. Iridoid glycosides in MO mainly include monotropein, asperuloside, deacetylasperuloside and deacetylasperulosidic acid, and the root of MO contains more than 2.0% of these iridoid glycosides [10]. In addition, these iridoid glycosides have been demonstrated to possess remarkable analgesic and anti-inflammatory effects based on animal and in cell-based experiments [11]. Monotropein decreased the writhing frequency in an acetic acid mouse writhing model, and attenuated ear swelling induced by xylene. Monotropein also inhibited LPS-induced mRNA expression of TNF-α and IL-1β in RAW264.7 cells and inactivated the NF-κB signaling pathway [12, 13]. Our preliminary investigation found that monotropein, asperuloside, deacetylasperuloside and deacetylasperulosidic acid could inhibit secretion of NO, PGE2 and TNF-α, and the expression of iNOS and COX-2 in LPS-stimulated RAW264.7 cells, indicating that MO iridoid glycosides (MOIG) may be the active ingredients underlying the anti-inflammatory effect [6]. Therefore, we proposed hypothesis that MOIG possessed anti-inflammatory and anti-arthritic activities. The aim of the present study was to validate the effects of MOIG in anti-inflammation and anti-arthritis in animal experiments, and reveal the underlying mechanism of MOIG in LPS-stimulated RAW264.7 cells.

## Methods

### Reagents and apparatus

Reagents and instruments used in this study were High Performance Liquid Chromatography combined with Diode Array Detector (Agilent 1100 Series, USA); AG285 electronic analytical balance (Mettler-Toledo, Switzerland); Microplate Reader (Bio-Tek FLx800, USA); XDA-1 macroporous resin (LanxiaoTechnology Companies, Xi’an, China); Complete Freund’s Adjuvant (Sigma, USA; Lot SLBN5308V; PCode: 1002093124); indomethacin and methotrexate (China Pharmaceutical Co., Ltd., Shanghai); tripterygium glycosides (TGs, Xiahua Pharmaceutical Industry, Shanghai, China; Lot No.: 160903); Assay kits for IL-6, IL-1β, IL-17 and TNF-α (Ebioscience, USA). Fetal bovine serum (FBS), α-Modified minimal essential medium (α-MEM), phosphate buffered saline (PBS) and penicillin/streptomycin were obtained from Gibco company (USA). Dimethyl sulfoxide (DMSO) was purchased from WAK-Chemie Medical GmbH (Steinbach, Hesse, Germany). Antibodies against IKBα, JUNK, P-ERK, ERK and p-38 were purchased from Boster Biological Technology (Wuhan, China). All the other antibodies used in this study were obtained from Cell Signaling Technology (Beverly, MA, USA). Lipopolysaccharide (LPS) from *Escherichia coli* 055:B5 was purchased from Sigma-Aldrich Company (USA). The BCA kit for assay of protein was purchased from Biyotime (Shanghai, China).

### Preparation of iridoid glycosides from MO root

MO roots were purchased from a local herbal drug store in Guangdong Province of China, and authenticated by Prof. Q.Y. Zhang of Zhejiang University of Traditional Chinese Medicine (Hangzhou, China). The voucher specimen (MO 20180508) was deposited in the herbarium of this Department. MO extracts and MOIG were prepared as follows: MO roots (10 kg) were extracted by percolation with 160 L solution of ethanol-water (70:30, *v*/v) for 20 h, and then filtrated. The combined filtrate was concentrated under reduced pressure to obtain the MO extract. Then, the MO extract was diluted with water to obtain 1.0 g crud drug /mL working solution. MOIG were prepared as follows: MO roots (10 kg) were extracted by percolation with 70% ethanol for 16 h × 3. After adjusting the pH value to 4.5 with HCl solution, the extracts were concentrated to1.0 g crude drug/mL and adsorbed to the XDA-1 macroporous resin column overnight. The macroporous resin column was eluted with 1:10 distilled water, and then with 10% ethanol for 10 times. The elutes of 10% ethanol were concentrated with vacuum pressure to get MOIG. The content of iridoid glycosides in MO was analyzed by HPLC-DAD chromatography (Agilent 1100 series, USA) method. The Venusil MP Cl8 column (250 mm × 4.6 mm, 5 μm) was used to perform HPLC separation, and the mobile phase was consisted of acetonitrile (A)-0.2% phosphoric acid and 0.01 disodium hydrogen phosphate buffer salt (B), and the gradient program were as follows: 0–12 min, 1–2% A; 12–30 min, 2–25% A. The 235 nm wavelength was set to detect chromatographic peak. The flow rate of mobile phase was set at 1.0 mL/min, the column temperature was 25 °C, and the injection volume was at 20 μL.

### Animals

Kunming male mice weighing 18-20 g were purchased from Lingchang Biological Technology Co., Ltd., shanghai, China, and Wistar male rats weighing 180-200 g were purchased from XipuerBikai Experimental Animal Co., Ltd., Shanghai, China. All animals were acclimatized for 1 week under the condition of temperature 24 °C ± 0.5 °C, 40–60% relative humidity and 12 h light-dark cycle before further experiments.

### Acute toxicity test

Considering that our previous investigation had not observed any toxic reaction of MOIG at dose of 2-10 g/kg. The maximum feasible dose method was adopted to evaluate its acute toxicity [14]. The MOIG were prepared into solution of 450 mg/mL in distilled water, which is the maximum concentration of MOIG. Forty male mice fasted for 12 h were equally randomized into two groups, including MOIG-treatment group and normal control group. The mice in MOIG-treatment group were administrated to MOIG solution with the volumes of 0.5 mL/10 g, which is the maximum volumes of administration for mice. The mice in normal control group were administered to equal amount distilled water. Thus the mice will be exposure to maximum feasible dose of MOIG (22.5 g/kg) to observe its toxicity. Behavioral changes and toxicity symptoms were monitored 2 h post administration of MOIG, and signs of delayed toxicity were further observed for 14 days. This animal protocols were approved by the Experimental Animal Ethic Committee of the Second Military Medical University (No. SMMU-Pharm-0004).

### Acetic acid-induced writhing test

Sixty mice were randomly divided into six groups according to body weight, including a model control group (distilled water), an indomethacin treatment group (4 mg/kg, positive control), a MO extract (3 g crude drug/kg) treatment group, and three MOIG (50, 100 or 200 mg/kg) treatment groups. The animals were orally administered with the vehicle, indomethacin, MO extract or MOIG for three days, and then intraperitoneally injected with 0.7% acetic acid at a dose of 0.1 mL/10 g after 60 min of the last administration. The number of abdominal contractions was recorded within 20 min. This animal protocols were approved by the Experimental Animal Ethic Committee of the Second Military Medical University (No. SMMU-Pharm-0011).

### The cotton-pellet granuloma experiment in rats

The 42 rats were randomly divided into six groups based on body weight, including a (distilled water) model control group, indomethacin (2.5 mg/kg, positive control) treatment group, MO extract (1.5 g crude drug/kg) treatment group, and 25, 50 or 100 mg/kg of MOIG treatment groups. The rats were intraperitoneally injected with 0.6 ml chloral hydrate (0.1 g/ml) and implanted with two cotton pellets (20 mg) into the left and right axillas subcutaneously. The rats were orally administered with the vehicle, indomethacin, MO extract or MOIG qd for 1 week, and then killed by inhalation overdose of ether. The cotton pellets were stripped and dried at 60 °C, and then weighed up. This animal protocols were approved by the Experimental Animal Ethic Committee of the Second Military Medical University (No. SMMU-Pharm-0019).

### Air pouch granuloma test in mice

The 60 mice were randomized into six groups based on their body weight, including a model control group (distilled water), an indomethacin (4 mg/kg, positive control) treatment group, a MO extract (3 g crude drug/kg) treatment group and three MOIG (50, 100 or 200 mg/kg) treatment groups. The mice were anesthetized by injecting 0.3 ml chloral hydrate (0.04 g/ml). The induction of air pouch granuloma was as follows: the air of 3 mL was subcutaneous hypodermically injected to induce the formation of a regular oval pouch, and then Complete Freund’s Adjuvant (CFA) of 0.5 ml was injected into the pouch within 24 h. When CFA was in full contact with the pouch wall, the mice were intragastrically administered with the designated vehicle or drugs for consecutive 7 days, and then mice were executed by inhalation of excessive ether. The pouch granulation tissue was carefully peeled off and dehydrated at 60 °C. The weight of the granuloma tissue was weighed. This animal protocols were approved by the Experimental Animal Ethic Committee of the Second Military Medical University (No. SMMU-Pharm-0025).

### CFA -induced arthritis test in rats

Eighty rats were equally randomized into following groups, ie, normal control group, model control group, methotrexate (MTX, 3 mg/kg, twice a week, positive control) treatment group, TG (5 mg/kg/day, also positive control) treatment group, MO extract (1.5 g crude drug/kg) treatment group, and three MOIG (25 mg/kg, 50 mg/kg and 100 mg/kg) treatment groups. The rats were subcutaneously injected 0.1 ml CFA through the root of the tail as the first immunization, and then followed by giving an injection of 0.05 ml CFA into the right hind metatarsal footpad after 7 days of the first injection as second booster immunization. The animals in the normal and model control groups were administered with an equal volume of the vehicle, and the other animals were treated according to the experimental protocol after 10 days of the first immunization. The rat body weight was weighed weekly throughout the experiment. Signs of arthritis were assessed weekly from day 1 of first immunization to day 28. The swelling and duration of paws combined with 5-point scale were applied to evaluate severity of arthritis, no sign is scored as 0; ankle/wrist with signs is scored as 1; ankle plus tarsal of the hind paw and/or wrist plus carpals of the forepaw with signs are scored 2; metatarsals or metacarpals with signs are scored 3, and entire hind or fore paw with severe signs are scored 4. The arthritic score of each rat was maximally set at 8, ie 4 points× 2 hind paws) [15].

The rats were killed by cervical dislocation on day 28. The spleen and thymus were peeled and weighed after washing in ice-cold saline and patting dry. The ratio of the weight of wet thymus or spleen to body weight (mg/g) was used to represent the thymus or spleen index.

The blood was obtained via the abdominal aorta, and centrifuged at 3000 rpm to collect serum; the serum was storied at − 20 °C until assay. The levels of IL-6, IL-1β and IL-17 in serum were measured according to the manufacturer’s protocols of ELISA kits.

The hind paws and knee joints of rats were stripped, and then fixed in 10% neutral formalin solution, and decalcified in EDTA solution at 4 °C for 30 days. The decalcified hind paws and knee joints were cut into 5-8 μm slices, and stained with hematoxylin and eosin (H&E) for histological examination. Histopathological changes were observed under a light microscope. This animal protocols were approved by the Experimental Animal Ethic Committee of the Second Military Medical University (No. SMMU-Pharm-0040).

### Anti-inflammation analysis in vitro

RAW murine macrophage 264.7 cells were purchased from the Shanghai Institute of Basic Medical Sciences Cell Resource Center, and cultured in DMEM medium containing 10% FBS, 1% penicillin/streptomycin, at condition of 37 °C and 5% CO_2_ humidified air. The following experiments were carried out when RAW 264.7 cells confluence to 80%.

The MTT method was used to determine the viability of the cultured RAW 264.7 cells. Cells of 100 μL (3 × 10^4^cells/mL) were cultured in 96-well plates for 24 h, and then treated with MOIG at dose of 6.25, 12.5, 25, 50 and 100 μg/mL for another 48 h. The 150 μL serum-free α-MEM and 20 μL MTT (5 mg/mL) were placed into culture well of plates before the 4 h of end of the experiment, and cultured at 37 °C. The supernatant was gently aspirated and vortexed for 15 min after addition of DMSO. The absorbance at 540 nm was measured with a microplate reader.

RAW 264.7 cells of 1 mL (5 × 10^5^ cells/mL) were seeded into 24-well plates, and incubated for 24 h at 37 °C in 5% CO_2_. The cells were stimulated with LPS of 10 μg/mL for 4 h, and then administered with MOIG at a final dose of 25, 50 and 100 μg/mL for 24 h. Then, the supernatant (100 μL) was transferred into a 96-well plate, and 50 μL Griess reagent A and B solutions were added respectively. Finally, the amount of NO released was determined at 540 nm. The levels of TNF-α, IL-1β and IL-6 released into the medium were detected using corresponding ELISA kits.

RAW 264.7 cells of 2 mL (1 × 10^6^ cells/mL) were seeded into 6-well plates, and incubated for 24 h at 37 °C in 5% CO_2_. The cells were administered with MOIG at a final dose of 50 and 100 μg/ mL while stimulated with LPS of 10 μg/mL for 20 h. The cells were lysed and the protein content was determined using a BCA protein kit. The cell lysates were incubated with a sample buffer of 2 × Laemmli at 100 °C for 10 min. An equal amount of protein sample was separated by 10% SDS-PAGE and transferred to PVDF membranes. The membranes were blocked with 5% BSA for 2 h and then incubated with corresponding primary antibodies overnight at 4 °C, and then washed 3 times with TBST, incubated with the HRP-conjugated secondary antibody for 1 h at room temperature. The protein bands were detected with electrochemiluminescence (ECL) reagent, and scanned by E-Gel Imager (Tanon-5200 Multi, Shanghai, China).

### Statistical analysis

The SPSS 21.0 statistical software was used to analyze the experimental data. The application of analysis of ANOVA and Student’s t-test were to determine significant differences in experimental group data. Data are expressed as the mean ± SD and the value of *p* < 0.05 was considered as statistically significant.

## Results

### Preparation of MOIG

**Fig. 1:**
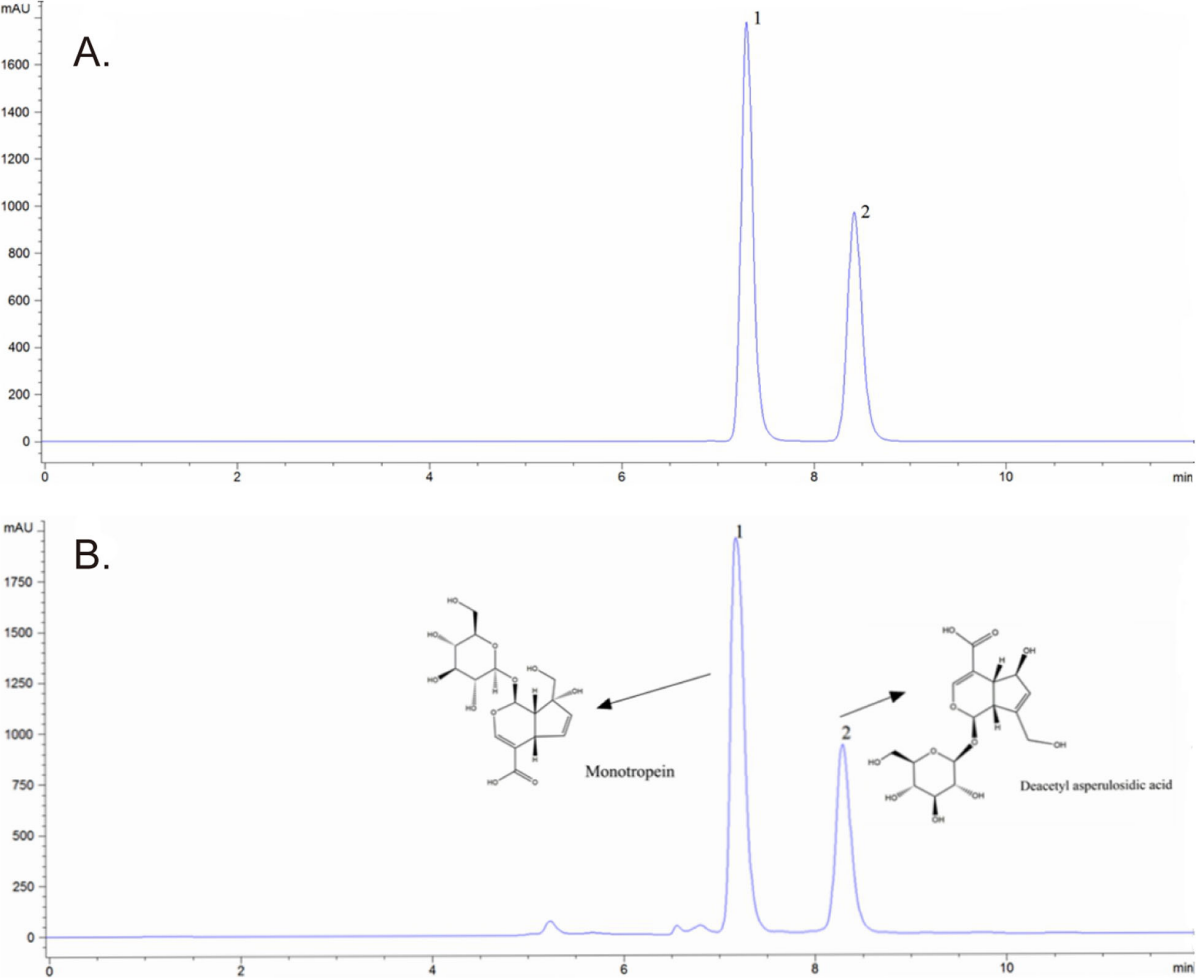
HPLC analysis of MOIG. **a** HPLC chromatogram of standard reference; **b** HPLC chromatogram of MOIG. Peaks were detected at 240 nm. 1: monotropein; 2: Deacetyl asperulosidic acid

The MOIG were enriched through XDA-1 macroporous resin, and 265 g MOIG were obtained from 10 kg root of MO. The results of HPLC analysis of MOIG are shown in Fig. 1. The results of HPLC analysis of MOIG are shown in Fig. 1. The content of deacetyl asperulosidic acid and monotropein in MOIG was calculated as 25.9 and 35.9% respectively. The content of monotropein and deacetyl asperulosidic acid in the MO was 1.24 and 0.73%, respectively.

### Acute toxicity test of MOIG

MOIG were administered to mice at maximum feasible dose of 22.5 g/kg to observe its acute toxicity. The observed toxicity of MOIG at dose of 22.5 g/kg was mainly mild sedation and reduced motor activity, which recovered to the normal state after 1 h of MOIG administration. Otherwise no toxic reaction or death of mice were observed during the 14-day observational period. These results indicated that MOIG had no significant toxicity, and exhibited a good safety.

### Analgesic and anti-inflammatory effects of MOIG in vivo

**Fig. 2:**
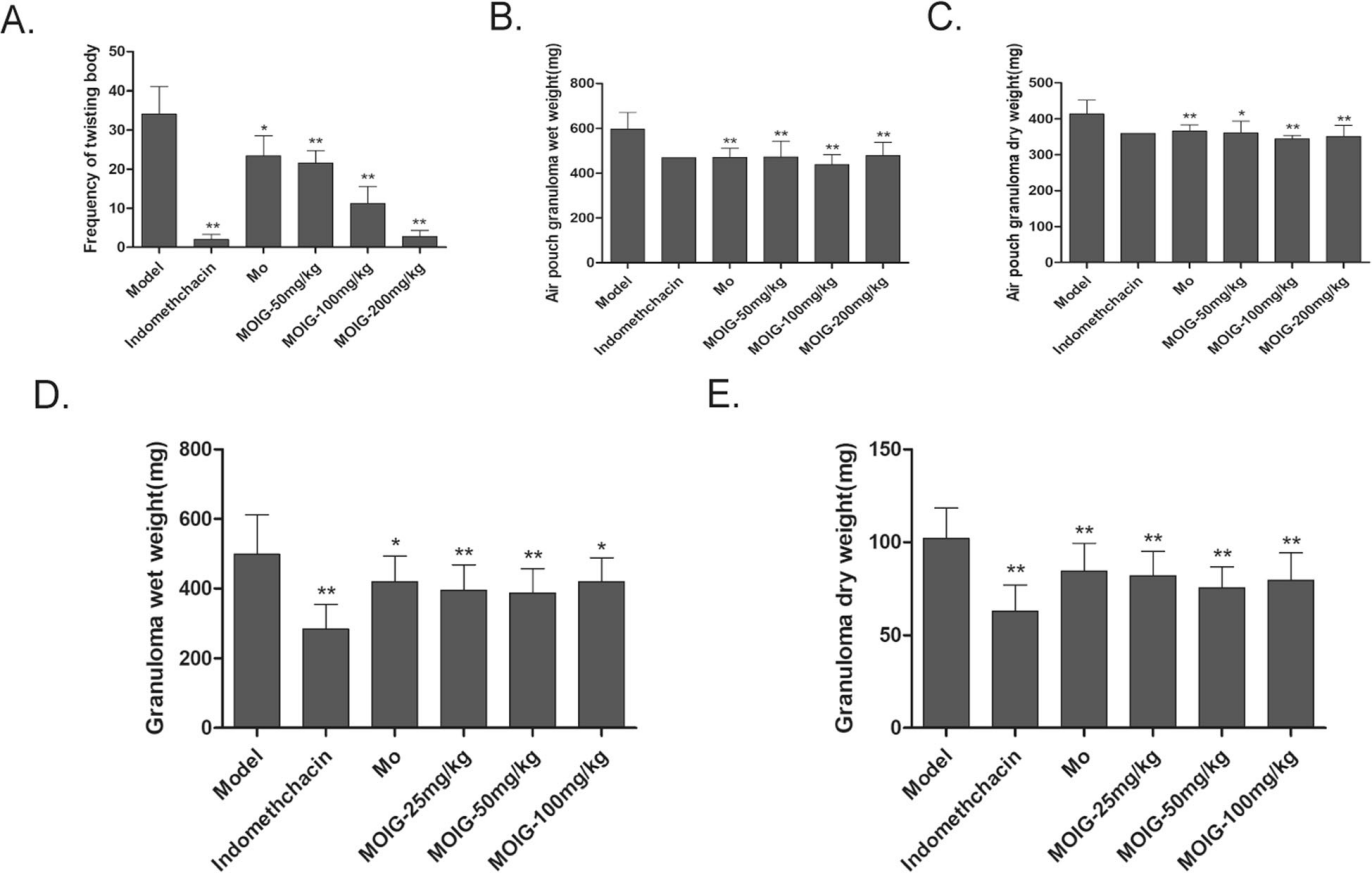
Analgesic and anti-inflammatory effects of MOIG. **a** MOIG inhibited the twisting induced by acetic acid in mice (*n* = 10); **b** and **c** MOIG attenuated inflammatory response as evidenced by wet weight and dry weight of air pouch granuloma in mice (*n* = 14); **d** and **e** MOIG mitigated inflammatory response as evidenced by wet weight and dry weight of cotton pellet granuloma in rats (*n* = 14). Data are presented as the mean ± SD. *p < 0.05, **p < 0.01, compared with the model control group

As shown in Fig. 2a, administration of the MO extract and MOIG at dose of 50,100 and 200 mg/kg, similar to that of indomethacin, exerted a dose-dependent anti-nociceptive response compared to model control group, as represented by a significant decrease in the frequency of writhing induced by acetic acid. The results of inflammation test showed that the MO extract and MOIG reduced the dry and wet weight of the air pouch granuloma in the mice at doses of 50-200 mg/kg (Fig. 2b and c), and also significantly decreased the dry and wet weight of the cotton pellet granuloma in the rats at doses of 25-100 mg/kg compared to model control group (Fig. 2d and e).

### MOIG improve arthritis symptoms, increase body weight in CFA -induced arthritic rats

**Fig. 3:**
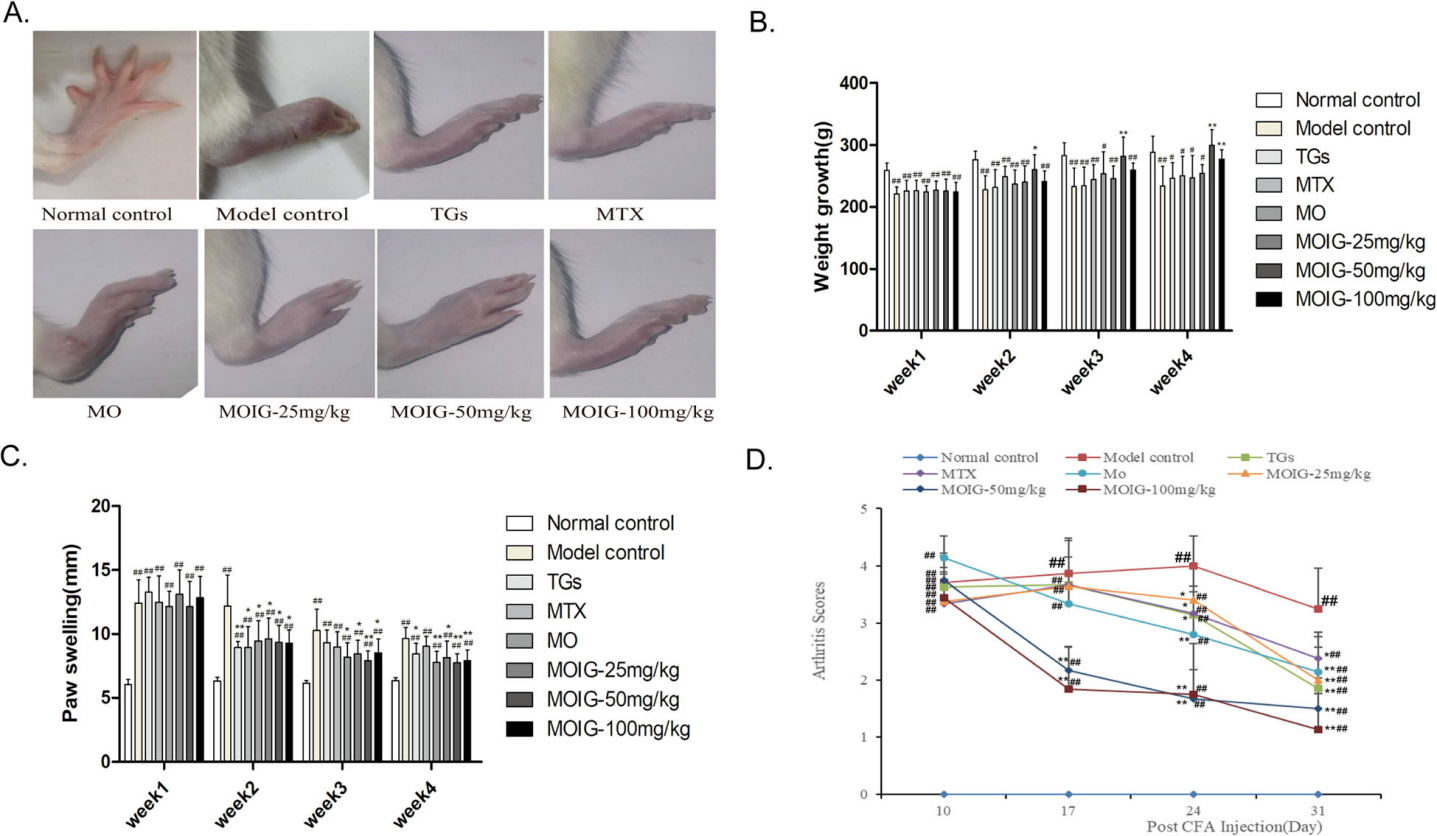
Effects of MOIG on CFA -induced arthritic rats. **a** Appearance of paw of CFA -induced arthritic rats; **b** Body weight; **c** Paw swelling (mm); **d** Arthritis score. Data are presented as the mean ± SD (*n* = 10). ^##^*p* < 0.01, compared with normal control group; *p < 0.05, **p < 0.01, compared with model control group. Note: Normal control, normal non-arthritic rats; Model control, arthritic non treated rats; TGs, Rats with arthritic and treated with TGs; MTX, Rats with arthritic and treated with MTX; MO, Rats with arthritic and treated with MO; MOIG-25 mg/kg, Rats with arthritic and treated with MOIG at dose of 25 mg/kg; MOIG-50 mg/kg, Rats with arthritic and treated with MOIG at dose of 50 mg/kg; MOIG-100 mg/kg, Rats with arthritic and treated with MOIG at dose of 100 mg/kg

The mitigating effects of MOIG on RA symptom were investigated in CFA -induced arthritic rats. The Fig. 3 showed that rats in model control group appear paw swelling and erythema, increased arthritis indices and significant weight loss, while both MTX and TG treatment (positive control) significantly improved the paw swelling, erythema and decreased arthritis indices, but had no effects on weight loss of the arthritic rats induced by CFA. MOIG treatment significantly mitigated paw swelling and the reduced arthritis indices at doses of 50–100 mg/kg, and slightly improved RA symptoms at a dose of 25 mg/kg. It was interestingly to find that treatment with MOIG at doses of 50 and 100 mg/kg could significantly enhance body weight of the arthritic model rats. Although the symptoms of arthritic model rats treated with the MO extract were also improved, the effects of MO treatment were not significant as compared with arthritic model group.

### MOIG decrease the spleen index but not the thymus index in CFA -induced arthritic rats

**Fig. 4:**
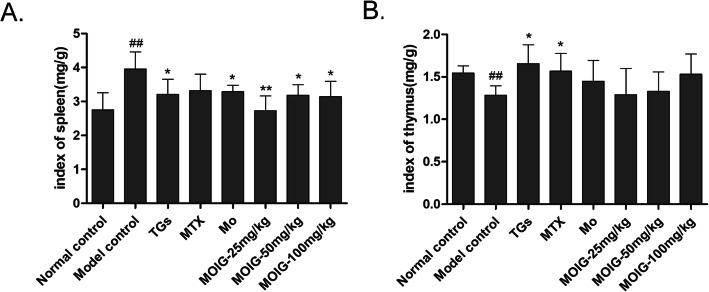
Effects of MOIG on the thymus index and spleen index of CFA -induced arthritic rats. **a** spleen index, **b** thymus index. Data are presented as the mean ± SD (*n* = 10). ^##^*p* < 0. 01 compared with the normal control group. *p < 0.05, **p < 0. 01 compared with the model control group. Note: Normal control, normal non-arthritic rats; Model control, arthritic non treated rats; TGs, Rats with arthritic and treated with TGs; MTX, Rats with arthritic and treated with MTX; MO, Rats with arthritic and treated with MO; MOIG-25 mg/kg, Rats with arthritic and treated with MOIG at dose of 25 mg/kg; MOIG-50 mg/kg, Rats with arthritic and treated with MOIG at dose of 50 mg/kg; MOIG-100 mg/kg, Rats with arthritic and treated with MOIG at dose of 100 mg/kg

As shown in Fig. 4, CFA induced a marked increase in the spleen index, and a significant decrease in the thymus index of rats in model control group, while MTX and TG (positive control) reversed the changes in the spleen and thymus indexes in arthritic model rats. The MO extract and MOIG decreased the spleen index but did not affect the thymus index of CFA -induced arthritic rats.

### MOIG reduce levels of inflammatory cytokines in serum of CFA -induced arthritic rats

**Fig. 5:**
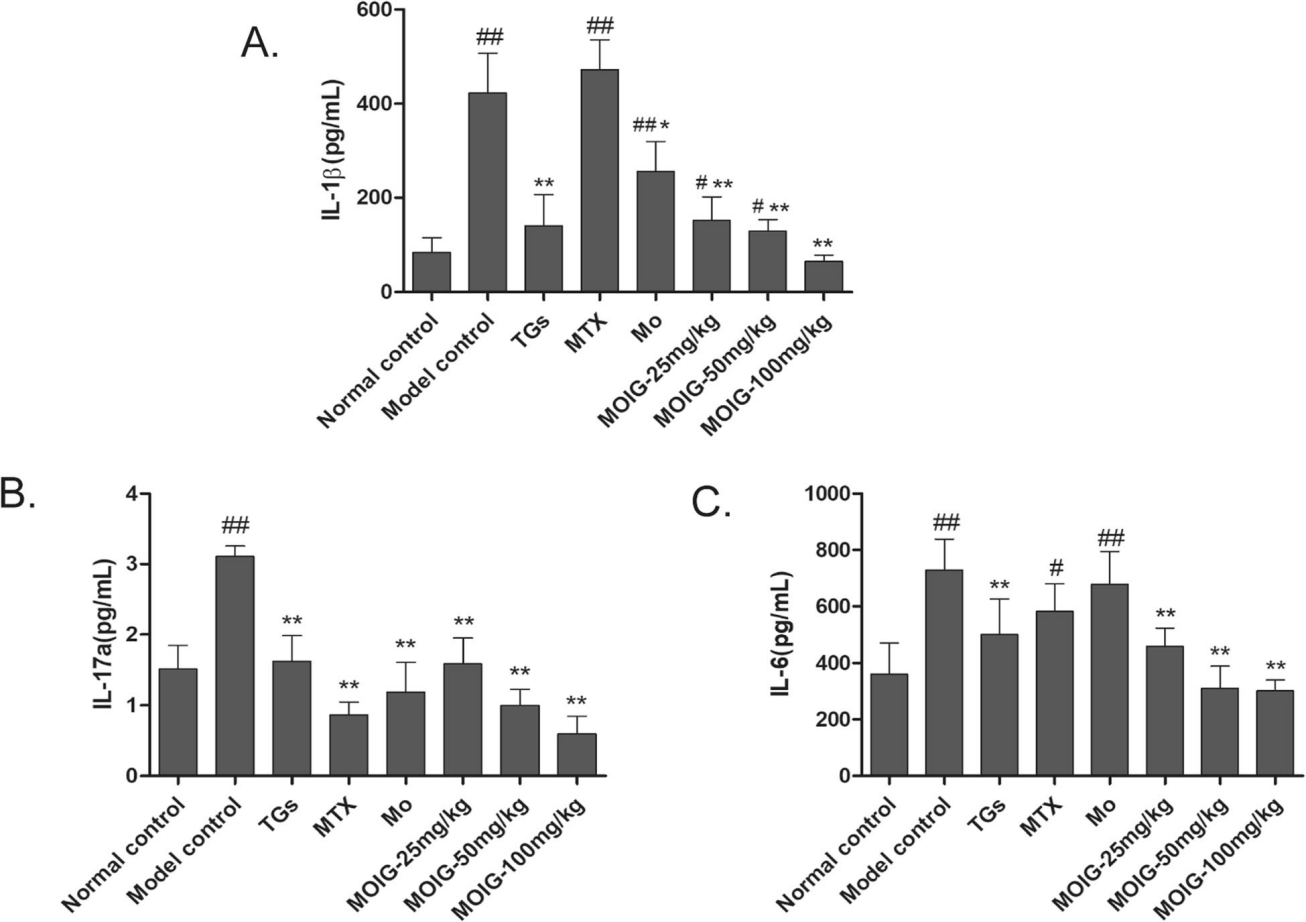
Effects of MOIG on serum levels of IL-1 β, IL-6 and IL-17a in CFA -induced arthritic rats. **a** IL-1β, **b** IL-17α, **c** IL-6. Data are presented as the mean ± SD (*n* = 10). ^#^*p* < 0.05, ^##^*p* < 0. 01 compared with the normal control group; *p < 0.05, **p < 0. 01 compared with the model control group. Note: Normal control, normal non-arthritic rats; Model control, arthritic non treated rats; TGs, Rats with arthritic and treated with TGs; MTX, Rats with arthritic and treated with MTX; MO, Rats with arthritic and treated with MO; MOIG-25 mg/kg, Rats with arthritic and treated with MOIG at dose of 25 mg/kg; MOIG-50 mg/kg, Rats with arthritic and treated with MOIG at dose of 50 mg/kg; MOIG-100 mg/kg, Rats with arthritic and treated with MOIG at dose of 100 mg/kg

Knowing that cytokines such as IL-1β, IL-6 and IL-17a play important roles in the pathogenesis of RA, their secretions were analyzed using ELISA kits. As shown in Fig. 5, the levels of pro-inflammatory cytokines IL-1β, IL-6 and IL-17a were significantly elevated in serum of rats in model control group in comparison with those in the normal control group. TG treatment decreased the levels of IL-1 β, IL-6 and IL-17a, and MTX treatment reduced the serum level of IL-17a, while MOIG treatment at doses of 25, 50 and 100 mg/kg abated levels of IL-1 β, IL-6 and IL-17α in serum of CFA -induced arthritic model rats.

### MOIG mitigate hyperplasia of the synovial tissue in CFA -induced arthritic rats

**Fig. 6:**
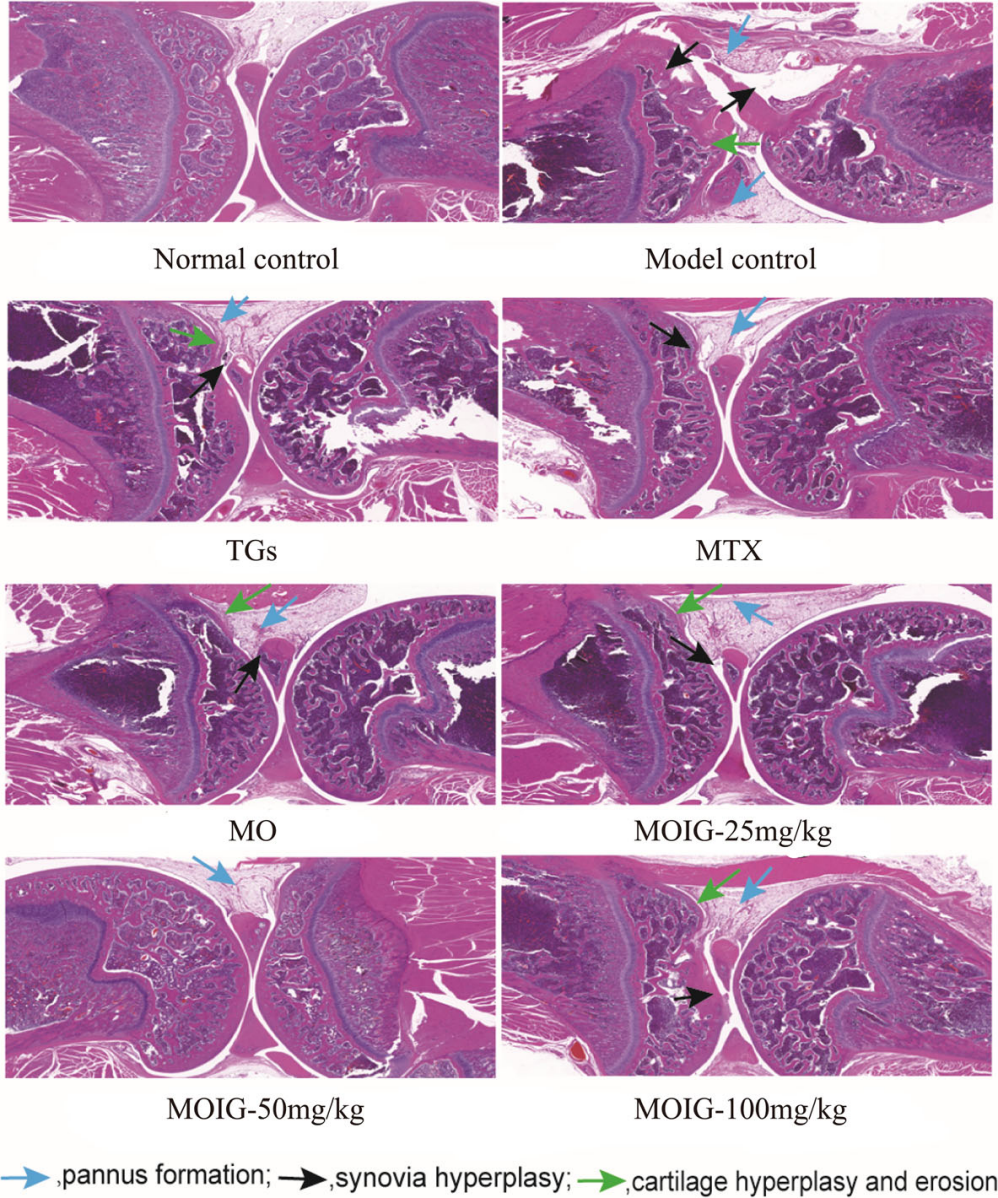
Effects of MOIG on the histopathological characteristics of joint and synovial tissue in CFA -induced arthritic rats (HE, × 200). CFA -induced arthritic rats exhibited severe synovial hyperplasia, infiltration of inflammatory cells, and pannus formation, which severely lead to cartilage hyperplasia and erosion. TGs, MTX, MO and MOIG reversed these pathological alterations in joint and synovial tissue of CFA -induced arthritic rats. Note: Normal control, normal non-arthritic rats; Model control, arthritic non treated rats; TGs, Rats with arthritic and treated with TGs; MTX, Rats with arthritic and treated with MTX; MO, Rats with arthritic and treated with MO; MOIG-25 mg/kg, Rats with arthritic and treated with MOIG at dose of 25 mg/kg; MOIG-50 mg/kg, Rats with arthritic and treated with MOIG at dose of 50 mg/kg; MOIG-100 mg/kg, Rats with arthritic and treated with MOIG at dose of 100 mg/kg

The histopathological results showed that the rats in normal control group are characterized with intact articular cartilage and normal joints and synovial tissues, while the rats in model control group manifested a severe bone deterioration and inflammation filtration in the joints and synovial tissues. Treatment with TG, MTX and MOIG significantly reduced inflammation filtration and bone deterioration in the articular and synovial tissues in CFA -induced arthritic model rats (Fig. 6).

### MOIG inhibit secretion of inflammatory cytokine in LPS-stimulated RAW264.7 cells

**Fig. 7:**
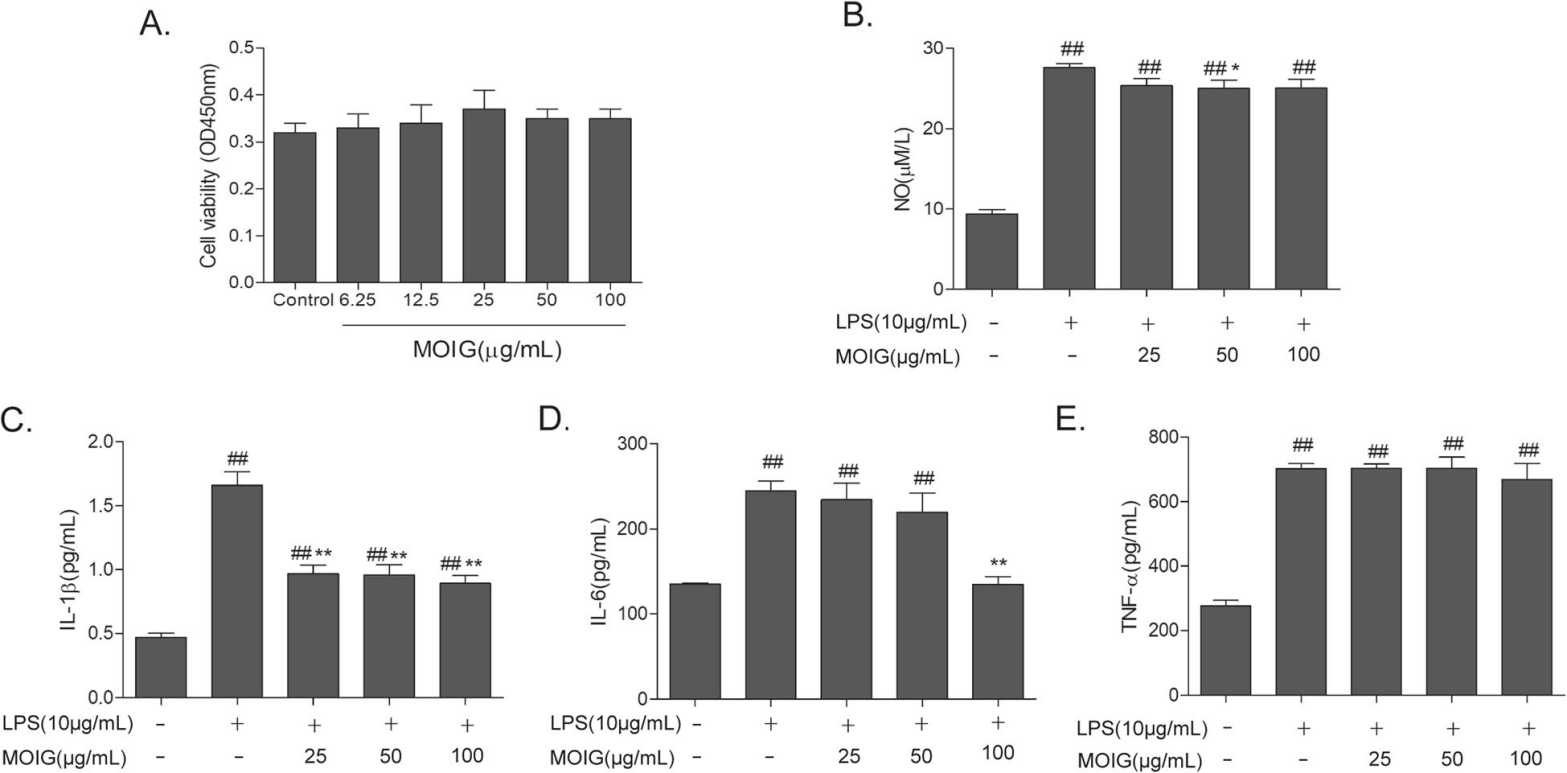
Effects of MOIG on the production of inflammatory cytokines in LPS -stimulated RAW264.7 cells. **a** cell viability; **b** NO level; **c** IL-1β; **d** IL-6; **e** TNF-α. Data are presented as the mean ± SD (*n* = 6) ^##^*p <* 0. 01 compared with the normal control group; *p < 0.05, **p < 0. 01 compared with LPS - stimulated control group

Furthermore, RAW264.7 cells were applied to evaluate the effect of MOIG on inflammatory cytokine production. As shown in Fig. 7, MOIG did not exert any effects on the viability of RAW264.7 cells at doses of 6.25-100 μg/mL. LPS treatment leads to a significant increase in release of NO, and levels of IL-1β, IL-6 and TNF-α in RAW264.7 cells. MOIG decreased the secretion of NO and IL-1β at doses of 25-100 μg/mL, and also inhibited the secretion of IL-6 at the dose of 50 μg/mL but did not affect the level of TNF-α at doses of 25-100 μg/mL in LPS-stimulated RAW264.7 cells.

### MOIG suppress iNOS and COX-2 expression in LPS-stimulated RAW 264.7 macrophages through inactivating the MAPK and NF-κB signaling pathways

**Fig. 8:**
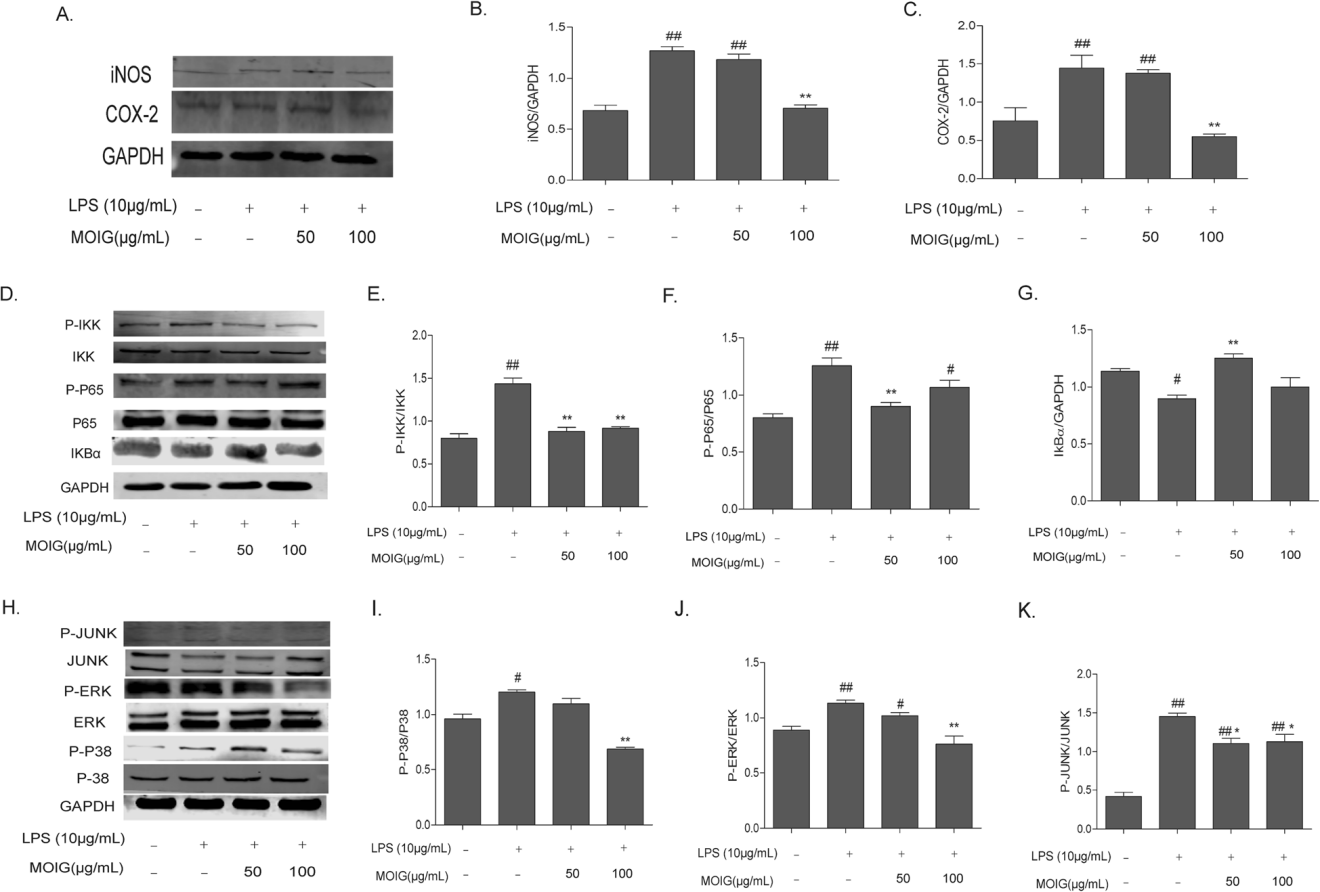
MOIG modulated the expression of COX-2, iNOS, and NF-κB and MAPK pathway in LPS -stimulated RAW264.7 cells. **a**, **b** and **c** Western -blot analysis for COX-2 and iNOS; **d**, **e**, **f** and **g** Western -blot analysis for related proteins of the NF-κB pathway; **h**, **i**, **j** and **k** Western -blot analysis for related proteins of the MAPK pathway. The experiments were repeated 3 times. Data are presented as the mean ± SD (*n* = 3) ^#^*p* < 0. 05, ^##^*p* < 0. 01 compared with the normal control group; *p < 0.05, **p < 0. 01 compared with LPS - stimulated control group

Knowing that iNOS and COX-2 is important mediator of inflammatory response induced with LPS, their expressions in LPS-stimulated RAW 264.7 cells were analyzed using western blot method to clarify the regulatory effect of MOIG. As shown in Fig. 8a, b and c, LPS stimulation markedly enhanced the protein levels of iNOS and COX-2 in RAW 264.7 cells, while MOIG treatment at a dose of 100 μg/mL significantly reduced the protein levels of iNOS and COX-2 in LPS-stimulated RAW 264.7 cells.

Furthermore, we investigated the molecular mechanism of MOIG anti-inflammatory through involving NF-κB and MAPK signaling pathways in LPS-stimulated RAW264.7 cells. In NF-κB pathway, treatment of RAW 264.7 cells with MOIG at dose of 50 and 100 μg/ml suppressed the elevation of the phosphorylation of IKK and p65 stimulated by LPS (Fig. 8d, e and f). In addition, MOIG increased the protein levels of IκB-α in LPS-stimulated RAW264.7 cells at dose of 50 μg/ml (Fig. 8d and g). In MAPK pathway, LPS stimulation increased the phosphorylation levels of JUNK, ERK and p38 in RAW264.7 cells, and MOIG treatment decreased their phosphorylation (Fig. 8h-k), suggesting that MOIG exerted anti-inflammatory effects through involving the NF-κB and MAPK signaling pathway.

## Discussion

The present study investigated the analgesic, anti-inflammation and anti-arthritic activities of MOIG. Considering that MOIG were prepared from MO, the anti-inflammatory and anti-arthritic activities of MO were investigated together with MOIG. The present study aimed to investigate the anti-inflammatory effects and mechanism of MOIG, which contains more than 50% iridoid glycosides, so only MOIG were explored the anti-inflammatory mechanism on RAW264.7 cells. The results showed that the MO extract and MOIG dose-dependently inhibited the twisting response in mice caused by acetic acid, decreased the dry weight and wet weight of cotton pellet granuloma in rats and air pouch granuloma in mice, and mitigated the symptom of paw edema of RA rats induced by CFA, indicating that MOIG possess anti-inflammatory and anti-arthritic effects. Further investigation on RAW264.7 cells indicated that the inhibitory effects of MOIG on inflammation may be related to their regulatory effects on the MAPK and NF-κB signaling pathways.

Iridoid glycosides from roots of *Morinda officinalis* mainly include monotropein, deacetylasperulosidic acid, asperuloside and asperuloside acid, and the contents of monotropein and deacetylasperulosidic acid are high, reaching to 1–2%, but the contents of asperuloside and asperuloside acid are too low, only reaching to 0.01–0.05% [16]. Therefore, asperuloside and asperuloside acid were not detected in MOIG. However, asperuloside and asperuloside acid, like as monotropein, also exert anti-inflammatory effects via suppression of the NF-κB and MAPK signaling pathways in LPS-induced RAW 264.7 cells, and can be used to treat obesity, inflammatory diseases, cancer and bacterial infections [13, 17]. Therefore, further research should be done to enrich the two chemical constituents in MOIG.

Acute toxicity studies in animals are usually necessary for any pharmaceutical intended for human use [14]. The traditional Chinese medicine and natural medicine often showed no or low toxicity, their LD50 values are often not deduced from multi-dose acute toxicity. The maximum feasible dose method was recommended to evaluate the toxicity of drug with low toxicity. The root of Morinda officinalis, which is often used in health food, exhibited no significant adverse effect and lower toxicity. And our preliminary investigation showed that MOIG at 2-10 g/kg showed no any toxic reaction. Therefore, maximum feasible dose method was used to evaluate its toxicity. In the present study, MOIG were administered to mice at maximum feasible dose of 22.5 g/kg to observe its acute toxicity, no toxic reaction or death of mice was observed during the 14-day observational period. These results indicated that MOIG had no toxicity, and showed a good safety.

The anti-nociceptive effect of MOIG was evaluated by acetic acid-induced writhing nociceptive experiment [18]. The acetic acid-induced pain response is due to the increased COX enzyme activity and PGE2 biosynthesis, and subsequently leading to an enhancement of arachidonic acid metabolites. Our previous study found that MO is involved in the regulation of arachidonic acid metabolism, and decreased the release of arachidonic acid metabolites in dexamethasone-induced osteoporotic rats [19]. Therefore, antinociceptive effect of MOIG might be due to the inhibition of the synthesis of arachidonic acid metabolites and the inhibitory effect on COX-2 activity.

The murine air pouch granuloma, which is similar to the synovium in morphology and functions, has been extensively used for the study of acute and chronic inflammation and inflammatory process [20]. The cotton pellet granuloma experiment is often applied to evaluate the inhibitory effects of drugs on chronic inflammation [21, 22]. The weight of wet cotton pellets represents the amount of exudative material, and the weight of dry pellets represents the amount of granulomatous tissue that correlated with the level of inflammatory and proliferative components [23, 24]. Our results showed that MOIG significantly reduced granuloma weight in murine air pouch model, decreased the dry weight of cotton pellet granuloma in rats, indicating that MOIG significantly mitigated acute and chronic inflammation response in vivo.

The CFA-induced arthritis model, which manifests as localized inflammation of the joints, hypertrophy of the synovial tissues and progressive and irreversible destruction of the cartilage, is similar to arthritis of human in serology and pathology, and often used to investigate RA pathogenesis and assesses potential therapeutics [25, 26]. The pathogenesis of CFA-induced arthritis includes two phases. The first and acute phase is the period of 0–10 days after CFA injection, in which the inflammatory response are mainly caused by various mediators released from leukocytes that provoke a vasculo-exsudatif phenomenon responsible for edema. The second and chronic phase is the period of 10–28 days after CFA injection, in which the inflammatory response are due to cellular inflammatory mediators, including IL-1β, IL-6, IL-17, TNF-α, interferon-γ and prostaglandins [26]. Paw swelling and arthritis index, which are featured with simple, sensitive and rapid determination, are commonly used to indicate the severity of arthritis and evaluate the anti-arthritic activity of drugs [3, 27]. In the present study, CFA -induced arthritic rats were treated with MOIG from day 10 to day 28 after CFA injection. Hence, the present study observed the effects of MOIG on the second phase of arthritis. It was found that both the diameter of the foot joint and the arthritic score were reduced in animals treated with *MOIG, as compared with* CFA -induced arthritic rats. This protective effect of MOIG was further confirmed by histopathology in our study, as represented by the reduced severity of synovial hyperplasia, cartilage and bone deterioration [28], exhibiting that MOIG significantly relieved the RA symptoms in CFA -induced arthritic rats.

It was reported that rat body weight was decreased during the progress of RA induced by CFA injection, and body weight change was often used as an indicator for evaluating the therapeutic effect of drugs [29]. The present study observed that the significant weight loss induced by CFA injection was mitigated after MOIG administration. The spleen and thymus are involved into the regulation of immune system, and RA is often complicated with obvious splenomegaly, splenitis and lymphoid hyperplasia. Hence, the relative weight of thymus and spleen are often used to estimate immune-regulatory activity of drugs against RA [3, 26]. The present study found that MOIG significantly reduced the spleen index, and did not affect the thymus index of CFA -induced arthritic rats, suggesting that MOIG may attenuated the symptom of RA through modulating the functions of immune organs.

Pro-inflammatory cytokines such as TNF-α and IL-1ß are known to play primary roles in mediating the pathological processes of inflammation and tissue destruction in RA. The elevated levels of pro-inflammatory cytokines and mediators aggravate the pain and deterioration of bone and cartilage. Moreover, TNF-α and IL-1β induce the expression of receptor activator of nuclear factor-κB (RANK) on macrophages and then promote these cells to differentiate into bone resorption osteoclasts [3]. Furthermore, IL-17 plays a synergistic role with TNF-α and IL-1β to amplify the inflammatory reaction. Therefore, RA can be prevented and treated through the strategy of suppressing the levels of these pro-inflammatory cytokines and mediators. The present study suggested that the levels of IL-1β, IL-6 and IL-17 was obviously increased in CFA -induced arthritic rats, and administration of MOIG could markedly and dose-dependently decrease their levels in serum of CFA -induced arthritic rats, indicating that MOIG attenuated the symptom of RA by reducing the inflammatory cytokine level [29, 30].

Activation of iNOS and COX-2 has been proven to play important roles in the pathogenesis of rheumatoid arthritis. It has been observed that iNOS and COX-2 catalyze the production of NO and prostaglandin E2 in inflammatory macrophages, and the prostaglandin E2 further induces secretion of IL-6, IL-1β and TNF-α, and then leading to severe inflammation responses [12]. This study showed that MOIG markedly inhibited NO release, reduced the protein expression of iNOS and COX-2, and decreased secretion of IL-1β, IL-6 and TNF-α in LPS-stimulated RAW 264.7 cells, further exhibiting that MOIG possessed definite mitigating effects on inflammation.

Excessive pro-inflammatory mediator production is mainly attributed to the activation of transcriptional factors during the inflammation progress. The stimulation macrophages with LPS activate NF-κB and MAPK pathways. The activation of NF-κB pathway causes the phosphorylation and degradation of IκBα, leading to NF-κB dissociation from the complex composed of NF-κB and (or) IκBα. The dissociated NF-κB p65 enter into the nucleus to modulate the expression and transcription of target gene related to inflammation [31, 32]. LPS stimulation also activates MAPK pathway, including ERK, JNK and p38, and the activation of MAPKs further promote IκBα phosphorylation, NF-κB activation and nuclear translocation, finally regulating the transcription of target gene [33, 34]. The present study showed that MOIG reduced the expression of p65, and also exerted significant inhibitory effects on p-ERK, p-JNK and p-p38, and phosphorylation and degradation on IκBα in LPS-stimulated RAW 264.7 cells, indicating that MOIG hindered the inflammatory response through controlling the MAPK and NF-κB signaling pathways.

In the present study, indomethacin, methotrexate (MTX) and tripterygium glycosides (TGs) were used as positive control in the animal experiments. Indomethacin, which is a nonsteroidal anti-inflammatory drug, exerts analgesic and anti-inflammatory activity by decreasing the production of prostaglandins. Methotrexate (MTX), which is an antifolate metabolite, has anti-inflammatory and immune-modulating properties. The anti-inflammation effects of MTX are involved into multiple aspects, inclusion of inhibition of purine and pyrimidine synthesis, promotion of adenosine release, and reduction of antigen-dependent T-cell proliferation [35–37]. TGs, which derived from botanical materials, exhibits immune-suppressive functions, and also attenuates inflammation and arthritis through reducing PGE2 production and levels of IL-1α, IL-1β, TNF-α and IL-6 [37]. Our investigation revealed that MOIG showed anti-inflammation and anti-arthritic activities and the underlying mechanism of MOIG maybe lie in its regulation on NF-κB and MAPK pathway.

## Conclusions

The results of our study showed that MOIG could effectively alleviate the symptoms of RA due to their potent anti-inflammatory and analgesic effects. These anti-inflammatory and analgesic activities of MOIG may be related to their abilities in inhibiting the frequency of twisting induced by acetic acid in mice and in decreasing the dry and wet weight of cotton pellet granuloma in rats and air pouch granuloma in mice. The anti-arthritic activity of MOIG is probably attributed to decreasing the spleen index and the serum levels of IL-1β, IL-6 and IL-17 in CFA -induced arthritic rats. Our study also indicated that MOIG exerted an anti-inflammatory action in LPS-stimulated RAW 264.7 macrophages by inhibiting the expression of iNOS, COX-2, IL-1β and TNF-α. We assume that these inhibitory effects of MOIG might be through inactivating the NF-κB and MAPK signaling pathways. Therefore, the present study strongly supports the potential of MOIG as a novel anti-inflammatory agent.

## Data Availability

All data sets used and/or analyzed during the current study are available from the corresponding author on reasonable request.

## Abbreviations

MO: *Morinda officinalis*
MOIG: *Morinda officinalis* iridoid glycosides
CFA: Complete Freund’s adjuvant
MTT: 3-(4, 5-dimethylthiazol-2-yl)-2, 5-diphenyl tetrazolium bromide
RA: Rheumatoid arthritis
HPLC: High-performance liquid chromatography-diode array detection
TGs: Tripterygium glycosides
MTX: Methotrexate
H&E: Hematoxylin and eosin
